# Seeking assistance in later life: how do older people evaluate their need for assistance?

**DOI:** 10.1093/ageing/afx189

**Published:** 2018-01-05

**Authors:** Krysia Canvin, Catherine A MacLeod, Gill Windle, Amanda Sacker

**Affiliations:** 1 University College London, England; 2 University of Bangor, Wales, UK

**Keywords:** qualitative research, older people, service uptake, needs evaluation, assistance

## Abstract

**Background:**

legislation places an onus on local authorities to be aware of care needs in their locality and to prevent and reduce care and support needs. The existing literature overlooks ostensibly ‘healthy’ and/or non-users of specific services, non-health services and informal assistance and therefore inadequately explains what happens before or instead of individuals seeking services. We sought to address these gaps by exploring older adults’ accounts of seeking assistance in later life.

**Methods:**

we conducted semi-structured qualitative interviews with 40 adults aged 68–95. We invited participants to discuss any type of support, intervention, or service provision, whether medical, social, family-provided, paid or unpaid.

**Findings:**

this paper reports older people’s accounts of how they evaluated their need for assistance. We found that the people in our sample engaged in a recursive process, evaluating their needs on an issue-by-issue basis. Participants’ progression through this process hinged on four factors: their acknowledgement of decline; the perceived impact of decline on their usual activities and independence; their preparedness to be a recipient of assistance; and, the opportunity to assert their need. In lieu of seeking assistance, participants engaged in self-management, but also received unsolicited or emergency assistance.

**Conclusions:**

older people’s adaptations to change and attempts to meet their needs without assistance mean that they do not present to services, limiting the local authority’s knowledge of their needs and ability to plan appropriate services. Our findings offer four stages for policymakers, service providers and carers to target to address the uptake of assistance.

## Introduction

The World Health Organisation’s concept of an ‘Age Friendly World’ [1] that helps people stay connected and healthy and provides support to those in need is being adopted across the UK. Legislation places an onus on UK local authorities to be aware of care needs and to prevent and reduce care needs in their locality (i.e. the Care Act 2014 in England, Social Services and Well-being Act 2014 in Wales). Challenges to achieving these aims include identifying people needing assistance before they require extensive care packages, and reaching non-users of specific services. Although considered heavy users of services older adults often do not take-up available health and social care due to barriers to access, denial of need or lack of information [2–5]. The extensive literature details these and other reasons (e.g. see Dixon-Woods *et al.*’s synthesis [6]). The usefulness of these studies in understanding how older people recognise and respond to emerging needs is limited, however, because they sample individuals with chronic health problems and/or existing service users, thus excluding ostensibly ‘healthy’ and/or non-users of specific services, non-health services and informal assistance. Similarly, the extensive health behaviour literature [7] informs our understanding of individuals’ uptake of services and treatments, but excludes non-health aspects of individuals’ lives and broader types of assistance [cf. 8, 9]. Consequently, the literature inadequately explains what happens *prior* to individuals’ contact with services or what they do instead of seeking assistance. In this qualitative study, we sought to address these gaps by exploring older adults’ experiences and views of assistance with health, social and other issues from services or other sources. Unlike other studies, we did not recruit participants based on their health status or service use. Given the policy emphasis on prevention, this paper focuses mainly on participants’ reasons for not seeking assistance from outside the household, what they did instead and the consequences.

## Methods

### Design

This qualitative interview study was part of a research programme examining older people and social exclusion utilising two ESRC-funded longitudinal datasets with common questionnaire items and geographical areas: UK Household Longitudinal Study (UKHLS) [10] and Cognitive Function and Ageing Studies Wales (CFAS Wales) [11]. The design is best described as ‘modified’ Grounded Theory [12–14].

### Sampling and recruitment

We acquired permission from the UKHLS hosts to conduct an ‘associated’ study then examined quantitative data from UKHLS and CFAS Wales to identify potential participants. We used purposive sampling [15] to identify a maximum variation subsample of individuals aged over 65 years living in North West England (UKHLS) and North Wales (CFAS Wales). We sought similar numbers of men and women, and individuals living with and without a partner. Recruitment procedures followed respective survey guidelines.

The UKHLS hosts sent invitations and information sheets to 80 respondents identified by the authors. Twenty interested individuals returned a reply slip which the UKHLS hosts passed to the authors. KC telephoned these individuals to answer questions and confirm agreement to participate. Of these, one was ineligible to participate but an additional respondent (a participant’s partner) volunteered. We expected and achieved a 25% response rate.

As CFAS Wales hosts, the authors sent information letters to 22 individuals inviting them to be interviewed, then telephoned them to answer questions and ask if they would like to participate. Two invitees declined (91% response rate).

### Data collection

KC (England) and CM (Wales) conducted semi-structured qualitative interviews in English in participants’ own homes. We digitally recorded, transcribed and anonymised all interviews. We used NVivo to store, retrieve and manage the combined datasets. The interview schedule comprised open-ended questions designed to elicit participants’ perceptions, experiences and views of needing assistance in later life but we encouraged participants to discuss topics of their choosing. To avoid arbitrary divisions between health, social and other types of assistance, we prompted participants to include any type of support, advice, intervention or service, whether medical, social, financial, housing or transport. We enquired whether assistance was provided by family, friends, neighbours, professionals or others and whether they paid or not. We probed participants’ accounts of how they identified ‘a need for assistance’, if, how, why (/why not) they acted on that perceived need, what they did instead and the consequences.

### Analysis

KC and CM jointly developed a coding framework by open-coding [13] their own interviews (maximising meaningful interpretation), double-coding and comparing several transcripts, and frequently discussing the data, codes and categories. We used the ‘constant comparison’ technique throughout [16]. KC and CM then combined the English and Welsh datasets and conducted thematic analysis [17] of different categories (e.g. ‘reasons for pursuing/not pursuing assistance’, ‘strategies for dealing with emerging issues’). We identified themes such as ‘self-management’. Examining the interrelationships between these categories (axial coding [13]) led us to develop the four-stage process of evaluation described in the findings. Our approach drew on Grounded Theory [13], combining the inductive–deductive cycles of data collection, analysis, hypothesis-testing and additional selective coding [14] to refine our development of the process.

## Findings

We interviewed 40 participants: 20 in North Wales (CFAS Wales) and 20 (UKHLS) in North west England, including four couples (Table 1). Participants’ ages ranged from 68 to 95 (mean age = 79). Participants’ unique identifiers indicate site (U = UKHLS, C = CFAS Wales), gender, age and living alone (A) or with a partner (P).

**Table 1.** Sample characteristics

Characteristics	England (UKHLS)		Wales (CFAS)		TOTAL	
	Participants (*n* = 20)		Participants (*n* = 20)		(*n* = 40)	
	Living alone (*n* = 10)	Living with someone else (*n* = 10)	Living alone (*n* = 9)	Living with someone else (*n* = 11)	Living alone (*n* = 19)	Living with someone else (*n* = 21)
Gender						
Male	4	6 (3)	4	6 (1)	**8**	**12 (4)**
Female	6	4 (3)	5	5 (1)	**11**	**9 (4)**
Age						
60s	0	0 (0)	0	2 (0)	**0**	**2 (0)**
70s	3	4 (2)	4	4 (0)	**7**	**8 (2)**
80s	5	4 (3)	3	3 (0)	**8**	**7 (3)**
90s	1	2 (1)	2	2 (2)	**3**	**4 (3)**
Not known	1	0 (0)	0	0 (0)	**1**	**0 (0)**
Marital status						
Married	2	8 (4)	0	10 (2)	**2**	**18 (6)**
Widowed	8	1 (1)	10	0 (0)	**18**	**1 (1)**
Divorced	0	1 (1)	0	0 (0)	**0**	**1 (1)**
Household						
Living alone	10	0 (0)	9	0 (0)	**19**	**0 (0)**
Living with spouse or partner	0	10 (6)	0	10 (2)	**0**	**20 (8)**
Living with spouse and children	0	0 (0)	0	1 (0)	**0**	**1 (0)**

Numbers given in brackets show how many participants took part in a couple interview.

**Figure 1.**
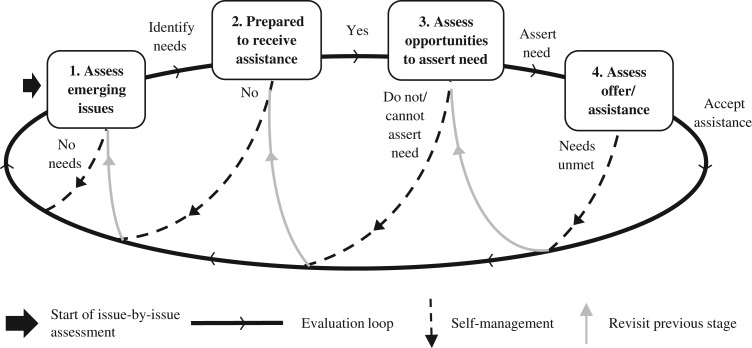
Process of evaluation of need for assistance.

Participants evaluated their need (and desire) for assistance on an issue-by-issue basis. Their considerations can be conceptualised as a recursive process comprising four stages (Figure 1).

### Stage 1. Assessment of emerging issues

Participants often perceived a high threshold for acknowledging needs requiring assistance: ‘If I can walk, I can work’ (U12M91A). Many described themselves as ‘healthy’ or ‘living a normal life’ but later revealed needs. Crucially, from participants’ perspectives, they were not *managing* or *denying* needs, because they did not *perceive* any needs. This was the case even for participants who described potentially risky circumstances, such as C14M74A who despite a recent stroke, several falls and being unable to get out of the bath, saw his walking stick as temporary and did not have or use any other assistance aids at home. One reason for this lack of perception is that over time, participants modified their expectations, behaviour and environment. Some viewed decline as inevitable consequence of ageing:

When the spring comes you find you can’t do things quite so easy as you could the last year. I guess you can’t do much about that, you’ve got to accept it. (C18M84P)

Others described lowering their housework and gardening standards. All described modifying behaviour to avoid triggering or aggravating issues, e.g. by generally slowing down, refraining from or restricting activities such as driving, hobbies, decorating and going out. They also described making modifications to their home and garden and responding to emerging issues by, e.g. using handrails, moving slowly on stairs, watching people’s lips when they talk, turning onto their knees to get out of the bath, redesigning gardens and/or kitchens to minimise maintenance and ease use. These adaptations—whether intentionally or not—preserved participants’ status quo and stymied emerging needs. Participants recalled reaching a tipping-point when they could no longer deny the deterioration of hearing, sight or mobility and considered assistance:

If you can’t hear what people are saying to you, you tend to get isolated. You tend to switch off. And then it would appear that you’re being rude, […] I couldn’t understand what they were saying, you see, so I had to take some action. (C20M70A)

### Stage 2. Preparedness to be a recipient of assistance

We found that even where participants acknowledged a need for assistance, they did not necessarily want to perceive themselves—or be perceived—as someone who needed, sought and received assistance. This presented a significant hurdle for many, especially as self-reliance was perceived as ideal:

You’ve got to help yourself, haven’t you? It’s no good relying on other people to do it. (C18M84P)

Self-funded assistance seemed to complement participants’ preference for self-reliance: many employed cleaners and gardeners, bought assistance aids (e.g. personal alarm system, stairlift, phone amplifier, walking frame, handrails, trolleys, bed guards, non-slip mats) and had paid for operations, hearing tests and hearing aids. Meanwhile, concerns about being a ‘scrounger’, ‘burden’ or ‘charity case’ deterred participants from seeking free or low-cost provision (e.g. from Social Services, Charities, NHS). U7F85P avoided returning to the doctors because, ‘I have that much, I feel embarrassed’. While participants varied in terms of their financial means, none described ruling out assistance due to cost, only principle or preference.

Participants’ individual perceptions of age were also a consideration: some declared they were ‘not old enough’ or ‘too old’ for certain types of assistance:

I think it’s admitting my age probably. ((laughs)) And that I can’t manage. No, I feel, you know, well, not that old, I don’t really want one of those things [personal alarm] around my neck. (C12F82A)

No, I wouldn’t bother [enquiring about a chiropodist]. I’m ninety. ((laughs)) (C13M90A)

Many said it was important to remain active and independent for as long as possible, especially those perceiving assistance as inevitable: ‘Why do it now when it will come anyway?’ (U12M91A). Others presented themselves as having always been independent, or a ‘do-it-yourself’ person, even preferring not to use tradesmen. Where participants perceived assistance as synonymous with inability to cope and compromising independence, they managed their own needs rather than assert a need for assistance.

When we asked participants under what circumstances they *would* be prepared to seek assistance, they indicated considerable reluctance: C10F86 said she would not use a wheelchair unless she was ‘desperate’; U12M91A said that he would only get help: ‘When I can’t walk or have gone ‘doodah’’. Participants revealed increased preparedness to receive assistance when they were unable to undertake their usual activities, i.e.: housework; household maintenance; climbing stairs; personal care; leaving the house (and how far they could go); socialising; and hobbies. Other tipping points included overwhelming pain, embarrassment, anxiety and loss of confidence:

It was perfectly level and I just tripped. So now I am a bit timid, you see and I feel a bit better with the stick. (U8M78P)

### Stage 3. Assessment of opportunities to assert need for assistance

Once amenable to receiving assistance, participants ranked their preferred ‘providers’. Overall, they reported preferring people they knew and recommended providers to ensure quality and reduce the risk of exploitation (not least due to negative past experiences). Such assurances were more common for those living in small, close-knit communities. When participants needed general assistance (e.g. minor household repairs, shopping, cleaning, gardening, transport) and even in emergencies they reported calling on ‘family first’ or less often, friends or neighbours:

I rang [my friend] and said, ‘[…] My nose is bleeding, it won’t stop.’ […] So she called the paramedics and then they said to me, ‘If it happens again you must call the paramedics straightaway instead of getting friends in to help you.’ (C13M90A)

Similarly, C18M84P called his son when he fell and did not see a GP afterwards because he was ‘fine’. For some, however, family assistance was limited or unavailable. Others preferred not to approach family, expressing concern about being a ‘burden’, ‘nuisance’ or causing worry.

Passivity was another factor: some participants expected to be told (e.g. by their doctor) if assistance were necessary:

My sister-in-law is saying, ‘Oh, you must have blood tests for this, blood tests for that,’ and I said, ‘He’ll [doctor] tell me if I need them.’ (C16F77A)

Opportunities were limited in other ways. A few participants described being unable to access services following closure or withdrawal (e.g. Post Offices, chiropodists, hearing clinics, milk/paper deliveries). Some—but not all—perceived that their age precluded certain types of surgery:

My doctor has said [a new hip] would be a bit of a risk anyway at my age. (C10F86P)

I should have open heart surgery but of course it’s too dangerous for them to do it at my age. (C6F94P)

I’ve never heard the words ‘you’re too old’. (U2M90P; awaiting shoulder surgery)

Others were unaware of potential assistance and how to acquire it: U5M82A did not know that hearing aids were available on the NHS, while C14M74A did not realise he could request a GP home visit or that charities offer home adaptations.

### Stage 4. Assessment of (potential) assistance

Sometimes, participants refused offers of assistance because of the perceived impact on independence or privacy:

I don’t want anybody pushing me [in a wheelchair] either. I value my independence. (C10F86P)

My daughter says, ‘Why don’t you get somebody to do the cleaning?’ I said, ‘I don’t want them in the house.’ (U3F87P)

They also expressed concern about ineffectiveness (e.g. the ‘flu vaccination) and risks. Others, however, simply did not want or like the assistance offered:

I don’t want another operation. (U7F85P)

[Carers were] trying to introduce these um dinners, you know, but I didn’t like those ((laughs)). (C2F90A)

Several participants reported that their needs remained unmet after accepting assistance. In such circumstances, participants revisited one or more previous stages in their evaluation, e.g. when C6F94P was dissatisfied with the telephone amplifier provided by Social Services she acquired a replacement from a charity.

In lieu of seeking assistance, participants engaged in self-management, but also received unsolicited or emergency assistance.

### Self-management

Participants reported managing without assistance in the absence of acknowledging needs, when they lacked preparedness to receive assistance or opportunity to assert their need and when they needed to compensate for the inadequacy or inappropriateness of assistance:

I think you have to do as much as you can on your own. I mean one day perhaps I’ll have to but as things go now I am still coping. (U14F75A)

My legs are giving me gyp [trouble] but I want to keep them going on the stairs as long as I can. Very important, yes, it is. I give it a good rub […]. I manage and that’s what matters. (U13F90A)

I’m still living in the paradise where I do everything myself if I wanted it. (U12M91A)

Self-management included actions described already, i.e. modifications made to behaviour, expectations and environment, and choosing consumer over service-user/patient status by purchasing assistance aids, pharmacy supplies and private services. Additionally, participants bypassed asserting a need for assistance by meeting their needs via alternative means. They reported using, e.g. gardening tools, shopping trollies, bedside tables for support when walking or standing and using assistance aids (e.g. personal alarm, bed guard) originally put in place for their partner. Some received support from a partner (sometimes reciprocated) and reflected how, if this ended, they would be unable to manage alone.

### Unsolicited interventions

Receiving assistance did not always depend upon participants seeking it but resulted from third parties intervening without invitation. These unsolicited interventions accelerated participants’ evaluations by overriding one or more stages. Participants revealed how relatives, friends, GPs and even passers-by had identified their need for assistance, and how unsolicited investigations and referrals arose from screening programmes, chronic health condition management, and unrelated visits to their doctor:

I went to the doctors […], I don’t know how it got about, but I said it’s a problem cutting your toenails as you get older. So I got this letter to go and see this, it wasn’t chiropodist, […] I went to the hospital […] and this lady came out and said, ‘Mr B, yes, come in, take your shoes and socks off, sit on the bed.’ And I said, ‘What are you going to do?’ She said, ‘I’m going to cut your toenails.’ I said, ‘If I’d known that I was coming in for my toenails, I’d have been embarrassed.’ […] She said, ‘Come back any time, just come in and make an appointment, we’ll attend to them.’ But I’ve managed on my own. (U19M81A)

Participants also described medical encounters where they received walking sticks/frames, personal alarms and ‘flu vaccinations that they would not otherwise have sought. Some perceived that age triggered these unsolicited interventions:

I think [GPs] have to see you every so often. When you get to a certain age they have to put a tick in the box, don’t they? (U3F87P)

Some unsolicited assistance was unwanted, e.g. C11F81A described feeling pressured into receiving an internet provider and C12F82A admitted misleading her children who encouraged her to carry her mobile in case she fell. Unsolicited assistance was viewed positively if unobtrusive, e.g. U12M91A said that the motion detector provided by his children was ‘great’ because he could ‘avoid people coming into the house’. Others described the benefits of discovering health conditions (e.g. osteoporosis, diabetes, prostate cancer) in this way.

### Crises and emergencies

Several participants reported receiving emergency care following the onset of infection, injury or stroke that, while not preventable might have benefitted from a speedier response or having a personal alarm, e.g. U13F90A had a stroke and was discovered by chance. Not everyone who had an accident, fall or other crisis sought assistance, or at least not immediately:

[I waited] a week, because I was, I started to lose blood which I hadn’t done before but it was only about five, six days say off my appointment so I thought I’ll just wait until I went in. (C17F72P with prolapse)

C15F73P asserted that she would seek assistance ‘if there’s something wrong’ yet delayed seeking medical assistance when she broke her ankle. Participants who described these events *did not* necessarily alter their subsequent behaviour to prepare for or minimise future incidents. U1M81A described several falls and had used his mobile telephone on one such occasion to contact his son, but no longer carried it and ‘never bothered about’ a personal alarm.

## Discussion

We found that our sample of older adults evaluated their need for assistance on an issue-by-issue basis, engaging in a four-stage recursive process. This process was not influenced by the type of issue arising, e.g. ill-health versus housework. Instead, participants’ progression through the process hinged on their acknowledgement of decline and its perceived impact on their usual activities and independence, their preparedness to be a recipient of assistance, and opportunities to assert their need. Participants told us that, in lieu of assistance, they employed a plethora of self-management techniques. They also revealed how, despite not seeking assistance, they received unsolicited and emergency assistance nevertheless.

Like Sarkisian *et al.* [2] we found evidence of age-related low expectations: our participants invoked age as a reason not to seek assistance (too old or not old enough) and to make sense of low expectations for assistance (to be expected ‘at my age’). While Dixon-Woods *et al.* [18] argue that the normalisation of ill-health symptoms coupled with low expectations for health made crises a route into services amongst deprived groups, low expectations only partly explain the behaviour of our sample. Some participants had high expectations for their abilities and led active lives: thus both high and low expectations drove participants’ reluctance to problematise everyday aspects of their lives. Instead, participants adapted to those changing circumstances by engaging in self-management, modifying their behaviour, environment and expectations (i.e. adapting to changes brought about by ageing [19]). Our findings illuminate how older people avoided and restricted a variety of activities and took alternative routes prior to formal (state-)provided assistance including calling on ‘family first’, paying privately for assistance and assistance aids or using furniture in place of rails or walking frames. These behaviours, however, inadvertently obscured and delayed the assertion of participants’ need for assistance, putting them at risk of crises and unsolicited interventions. Some participants living with their partner described co-dependency which too may have had a similar impact.

We identified many reasons why participants did not seek assistance from *services*—many of which have been described in the literature [4, 5]—but uniquely, we combine these with participants’ reasons for not pursuing assistance from *other sources*. The literature suggests that older people’s desire to preserve an independent, self-reliant, responsible self-image can deter assistance-seeking from outside the household [6, 9, 20]. Like others [21], we found that preparedness to receive assistance depended to some extent on participants’ notion of independence: assistance remained unpalatable while they could remain independent without it, but this view shifted where they perceived that independence could be achieved through assistance. Participants were especially concerned about the stigma of being someone who needs assistance, equating assistance with being a burden or scrounging which was incompatible with their self-image of being active and independent. They also expressed concern about exploiting free assistance, including that provided by family. Accordingly, they repeatedly indicated that asking for assistance was a last resort, regardless of provider. Other studies refer to the stigma of symptoms and conditions [22], but not stigma related to being a recipient of assistance *per se*.

There is some overlap between the process that we describe and other models. The Illness Action Model [23] proposes that individuals undertake evaluations and reassessment of their experiences, actions (and their potential impact) to attempt to restore ‘equilibrium’ to their lives. The Selective Optimisation with Compensation model [24] explains how people manage losses through successful adaptation or regulation of their behaviour, optimising assets and compensating for reductions in functioning. The concept of candidacy [18] encapsulates how individuals’ eligibility for and access to medical attention is determined by definitions of appropriateness which are influenced by socio-cultural, economic and organisational factors: individuals must perceive themselves as ‘legitimate candidates’ for a service before they ‘assert their candidacy’. Although the similarities with these models lend some credibility to our findings, the process we describe here is distinct in several ways. First, we derived our process from the analysis of a wide range of needs and types of assistance, not just health or social care. Second, although the candidacy model has been applied beyond healthcare [25, 26] it does not make explicit provision for individuals’ behaviour prior to or beyond their consideration of services. In contrast, our process acknowledges that prior to identifying oneself as a legitimate candidate for services, one must acknowledge a need for assistance *and* be prepared to be a recipient of assistance from any provider (including family). Third, our process illuminates unsolicited and emergency pathways into assistance. None of these models capture all these elements.

### Policy and practice implications

Understanding why older people do not seek or receive assistance is key to the development of policies and services that enable local government to meet their obligations to provide preventative care. Our findings illustrate how older people’s seemingly positive adaptations to changes in their abilities and attempts to meet their needs without assistance mean that they do not present to services with minor issues that provide opportunities for practitioners to identify other problems and offer unsolicited assistance. In this way, older people inadvertently limit local authorities’ knowledge of their needs and risks, making it difficult to plan appropriate services and interventions. Our findings also suggest that older adults’ reluctance to receive assistance or use assistance aids could prevent their uptake of preventative or even restorative assistance and put them at risk for emergency intervention. This is problematic if it means that preventative services are underutilised and resources are spent instead on crisis intervention.

The process that we describe here highlights four stages that policymakers, service providers and carers can target to address the uptake of assistance. Improving the availability and accessibility of assistance for older adults is important (Stage 3), but our findings suggest that attention must also be paid to Stages 1 and 2. It is at these earlier stages that interventions could target expectations, the stigma attached to receiving assistance, and preconceptions about independence and responsibility. Services could, for example, reframe assistance as promoting independence. Crucially, the recursive nature of the process where individuals constantly reassess emerging issues and needs present multiple opportunities for practitioners and others to offer assistance. This is particularly important given that individuals might not necessarily change their behaviour following a crisis or intervention and may be unwilling to voluntarily assert their need for assistance.

Our study is person-centred: rather than focusing on a specific condition or service, we examined older people’s accounts of all types of issues and assistance, whether provided via personal, public or private networks to deepen our understanding of these not as isolated decisions but as a process [7]. Our findings support the notion that service provision for older adults in the community should be ‘person-centred’ [20] despite the shortage of evidence [27, 28]. The transferability of our findings might be limited by our purposively sampled group of over-65s, who were selected regardless of health status or use of services. It is precisely this, however, that makes the study stand out from the existing literature and sheds light on aspects of service uptake that are so often overlooked.

**Key Points**

- Older people’s decisions about assistance involve considerations of decline, independence and preparedness to be a recipient.
- Older people might avoid assistance and treat public services as a last resort even in urgent circumstances.
- Older people’s attempts to self-manage their needs may put them at risk of unsolicited or emergency intervention.
- Services and carers must take account of older people’s reservations and preferences to improve the palatability of assistance.
